# Association between physical activity and sub-types of cardiovascular disease death causes in a general population cohort

**DOI:** 10.1007/s10654-018-0460-2

**Published:** 2018-11-11

**Authors:** Mark Hamer, Gary O’Donovan, Emmanuel Stamatakis

**Affiliations:** 1 School of Sport, Exercise and Health Sciences, Loughborough University, Loughborough, LE11 3TU UK; 2 Department of Epidemiology and Public Health, University College London, London, WC1E 6BT UK; 3 School of Medicine, Universidad de los Andes, Bogotá, Colombia; 4 Charles Perkins Centre Epidemiology Unit, University of Sydney, Sydney, Australia; 5 Prevention Research Collaboration, School of Public Health, University of Sydney, Sydney, Australia

**Keywords:** Physical activity, Cardiovascular diseases, Mortality

## Abstract

Physical activity is thought to be cardioprotective, but associations with different subtypes of cardiovascular disease (CVD) are poorly understood. We examined associations between physical activity and seven major CVD death causes. The sample comprised 65,093 adults (aged 58 ± 12 years, 45.4% men) followed up over mean [SD] 9.4 ± 4.5 years, recruited from The Health Survey for England and the Scottish Health Surveys. A CVD diagnosis was reported in 9.2% of the sample at baseline. Physical activity was self-reported. Outcomes were subtypes of CVD death; acute myocardial infarction; chronic ischaemic heart disease; pulmonary heart disease; a composite of cardiac arrest, arrhythmias, and sudden cardiac death; heart failure; cerebrovascular; composite of aortic aneurysm and other peripheral vascular diseases. There were 3050 CVD deaths (30.8% of all deaths). In Cox proportional hazards models adjusted for confounders, physical activity was associated with reduced relative risk of all CVD outcomes; compared with the lowest, the highest physical activity quintile was associated with reduced risk of acute myocardial infarction (Hazard ratio 0.66: 95% CI 0.50, 0.89), chronic ischaemic heart disease (0.49: 0.38, 0.64), pulmonary heart disease (0.48: 0.22, 1.07), arrhythmias (0.18: 0.04, 0.76); heart failure (0.35: 0.20, 0.63), cerebrovascular events (0.53: 0.38, 0.75); aneurysm and peripheral vascular diseases (0.54: 0.34, 0.93). Results were largely consistent across participants with and without existing CVD at baseline. Physical activity was associated with reduced risk of seven major CVD death causes. Protective benefits were apparent even at levels of activity below the current recommendations.

**Electronic supplementary material:**

The online version of this article (10.1007/s10654-018-0460-2) contains supplementary material, which is available to authorized users.

## Introduction

There is considerable evidence on associations between physical activity (PA) and reduced risk of cardiovascular disease (CVD) death [1–3], although the majority of these cohort studies have treated CVD as a composite endpoint and failed to consider the different sub-types. In larger samples it is common practise to investigate coronary heart disease [2] and stroke [3] as separate entities. However, less common CVD disorders (e.g., heart failure, cardiac arrest, ventricular arrhythmia, sudden cardiac death, abdominal aortic aneurysm, and peripheral arterial disease) have been rarely examined, particularly from within the same cohort. A direct comparison of effect estimates across sub-types of CVD may provide etiological insights about likely mechanisms of PA action on cardiovascular physiology. In addition evidence on PA and specific CVD outcomes is needed to develop more specific guidelines on prevention of subtypes of CVD.

Our objective was to examine the association between PA and seven major sub-types of CVD death in a large general population sample of adults living in the United Kingdom.

## Methods

Participants were recruited from 11 survey years of the Health Survey for England and the Scottish Health Survey; [4, 5] these included 1994 (HSE only), 1995 (SHS only), 1997 (HSE only), 1998 (HSE and SHS), 1999 (HSE only), 2003 (HSE and SHS), 2004, 2006, 2008 (HSE only).Local research ethics committees approved each survey and all participants gave written informed consent. PA in the 4 weeks prior to interview was assessed by a validated questionnaire [6]. PA was categorised into four groups: Inactive (participants not undertaking any PA of moderate or vigorous intensity); Insufficient activity (undertaking some moderate—vigorous PA but not meeting the current PA guidelines); Sufficient activity (those meeting and exceeding the guidelines 150 min/week moderate or 75 min/week vigorous PA); High activity (those exceeding 300 min/week moderate –vigorous PA) [1]. In addition we categorised total PA into quintiles based on metabolic equivalents (METs) per week, where one MET is considered to represent resting energy expenditure.

Individual participant data were linked with the British National Health Service Central Registry to record mortality. Audits have shown that these data are ~ 90% accurate in identifying the correct diagnosis, and completeness of data is ~ 99% [5]. Based on prior studies [7] we chose seven major CVD outcomes: acute myocardial infarction (ICD-10 code; I21); chronic ischaemic heart disease (I25); pulmonary heart disease (I26-28); a composite of cardiac arrest, arrhythmias, and sudden cardiac death (I46-49); heart failure (I50); cerebrovascular death (I60-I69); and a composite of aortic aneurysm and other peripheral vascular diseases (I71-73). Data for survivors were censored to 12/31/2009 (Scottish survey) or 3/31/2011 (English survey). Cox proportional hazards models were used to estimate associations of PA with CVD mortality. Based on prior work [1–3] models were adjusted for age, sex, smoking (never, previous, current), social occupational group (professional/managerial, skilled non-manual/manual, semi-skilled/non-skilled), presence or absence of chronic illnesses, psychological health (using the 12 item General Health Questionnaire). In sensitivity analyses we re-ran the models (on outcomes with sufficient numbers of events) after stratifying the sample into participants with (n = 5962) and without (n = 59,131) a physician diagnosis of CVD at baseline in order to explore possible reverse causation. All analyses were performed using SPSS version 22 (IBM Inc.).

## Results

The sample comprised 65,093 adults aged 40 years and above (aged 58 ± 12 years, 45.4% men). Physically active participants were younger, more likely to be male, never-smokers, from a higher social occupational group, report less illnesses (including prevalent CVD), and lower psychological distress (Table 1). There were 9900 deaths in total, 3050 of which were attributed to CVD (30.8% of all deaths) during 564,959 person-years of follow-up. Nearly half (48.5%) of all CVD was attributed to coronary heart disease (acute myocardial infarction and chronic ischaemic heart disease), with stroke (20.8%), heart failure (8.5%), aortic aneurysm and other peripheral vascular diseases (7.7%) pulmonary heart disease (2.8%), cardiac arrest, arrhythmias, sudden cardiac death (2.3%), and other causes (9.4%; too few events to separately analyse) making up the remainder.

**Table 1:** Characteristics of the sample at baseline

	Inactive (n = 40,413)	Insufficient activity (n = 13,121)	Sufficient activity (n = 6826)	High activity (n = 4751)
Age, years ± SD	60.6 ± 12.6	54.2 ± 10.6	52.5 ± 9.8	53.4 ± 10.3
Sex, % male	45.2	44.5	48.6	54.6
Cigarette smoking, %				
Never	41.4	49.3	53.4	51.0
Ex-smoker	32.9	31.7	31.6	33.0
Current	25.7	19.0	15.0	16.0
Occupation, %				
Professional/managerial	27.5	41.4	47.3	47.2
Skilled	44.6	40.9	36.7	36.1
Semi/unskilled	27.9	17.9	16.0	16.7
Chronic illness, %	60.2	45.4	40.3	38.7
Prevalent CVD^‡^, %	12.1	4.5	3.3	3.7
Psychological distress^†^, %				
Asymptomatic	57.8	64.6	68.8	72.6
Sub clinical	24.9	22.9	20.9	19.1
Symptomatic	8.3	6.5	5.6	4.4
High	9.0	5.9	4.7	3.9

^‡^Prevalent cardiovascular disease (CVD) defined as physician diagnosed angina, heart attack, stroke. ^†^Psychological distress divided into four groups based on scores from GHQ-12: asymptomatic (score = 0), sub clinically symptomatic (1–3), symptomatic (4–6), and highly symptomatic (7–12)

**Fig. 1:**
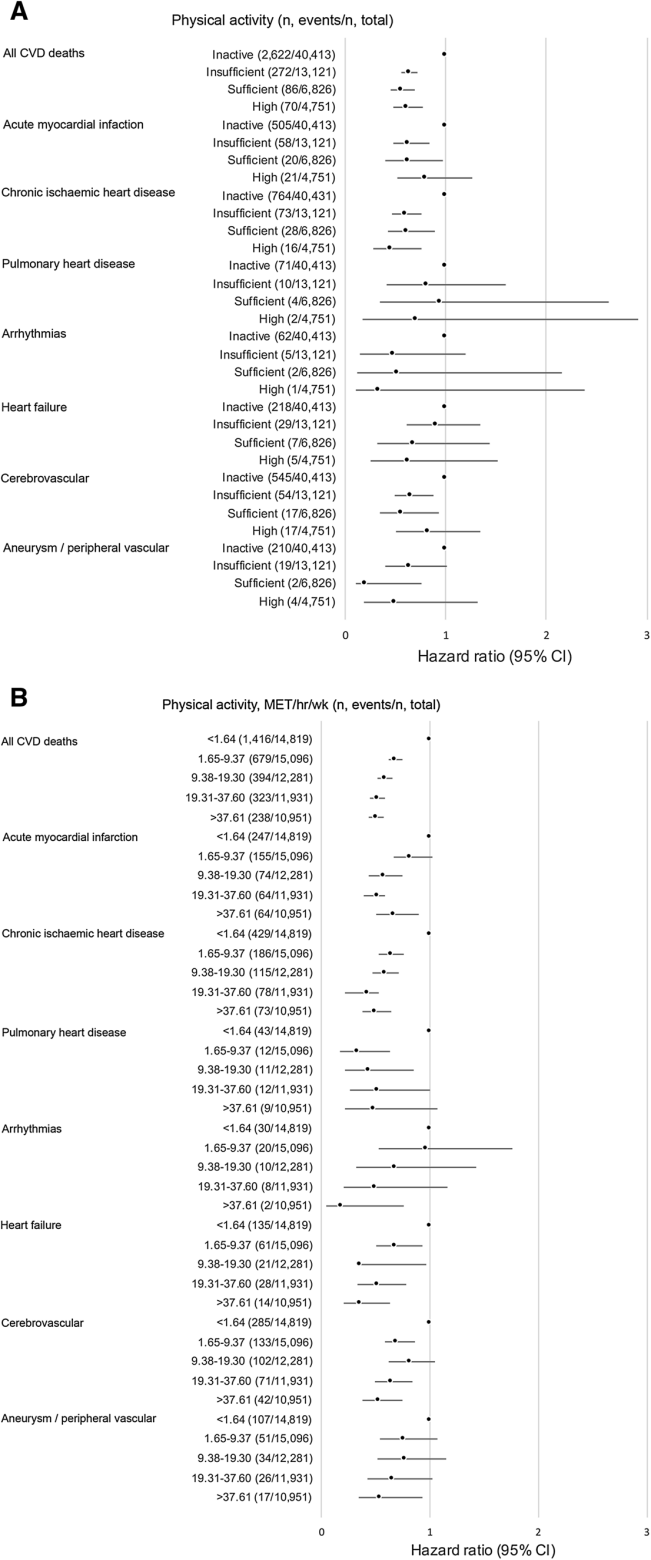
Associations between physical activity and different CVD death endpoints in participants aged 40 years and above in Health Survey for England/Scottish Health Survey (n = 65,093). **a** Categorises participants based on the physical activity guidelines; **b** Categorised total physical activity into quintiles based on metabolic equivalents (MET) hours per week. Hazard ratio (HR) adjusted for age, sex, smoking, social occupational group, chronic illnesses, psychological distress

Sufficient activity was associated with reduced risk of all CVD outcomes (Fig. 1a; Table e1 in ESM), albeit confidence intervals did not reach conventional levels of significance in outcomes with limited events (heart failure; pulmonary heart disease; cardiac arrest/arrhythmias/sudden cardiac death). Reduction in risk ranged from 17% (pulmonary) to 58% (aortic aneurysm/peripheral vascular diseases) and point estimates were remarkably consistent across coronary heart diseases, heart failure and cerebrovascular deaths, albeit, confidence intervals were wide and overlapping for some of the less common outcomes. Among participants meeting PA guidelines, around 41% exceeded them (300+ mins/week). In general, participants that exceeded the guidelines did not demonstrate noticeably greater risk reduction (compared to those meeting basic guidelines) (Fig. 1a). We did not find any evidence of interactions by sex.

We explored the shape of the association in more detail using total PA as a continuous variable (MET-h-week) split into quintiles (Fig. 1b). There was some evidence of dose–response relationships albeit, confidence intervals were wide and overlapping for some of the less common outcomes.

A CVD diagnosis was reported in 9.2% of the sample at baseline. Results were largely consistent across participants with and without existing CVD at baseline (Table e2 in ESM), except there was no association between PA and aortic aneurysm/other peripheral vascular diseases in participants with existing CVD.

## Discussion

The majority of large epidemiological studies have focused on associations between PA and a composite CVD endpoint or coronary heart disease [2]. Our objective was to examine the association between PA and different sub-types of CVD death. Physical activity was associated with reduced risk of all types of CVD outcomes that we examined. The associations appeared stronger when our exposure was based on total PA (not just restricted to moderate—vigorous intensity PA) suggesting that all types of PA may contribute to CVD prevention.

Consistent with our findings, an association was observed between PA and heart failure in a recent meta-analysis of 12 prospective studies [8]. There has been limited work on PA and prevention of peripheral vascular diseases, particularly in general population cohorts, although existing data from patient samples are consistent with our findings [9]. Findings on PA and arrhythmias has been mixed; recent studies have shown associations between PA and physical fitness with reduced risk of atrial fibrillation [10, 11] although others have suggested a non-linear pattern of results [12, 13]. Our data showed an inverse association between PA and risk of stroke although we were unable to further sub-type cerebrovascular events. Recent data have suggested curvilinear associations for PA and haemorrhage stroke, indicating protection at moderate PA levels and possible increased risk at very high levels of activity; in contrast very high levels of PA was not associated with increased risk of ischemic stroke in Asian populations [3, 14], although results may differ in Western cohorts [15]. Our findings on coronary heart disease suggested a plateau in risk reduction at higher levels of PA. Nevertheless, recent work [11] comparing associations of self-reported and objective PA on CVD risk observed curvilinear and linear curves, respectively, suggesting that reporting bias might possibility explain increased risk in participants that mis-report very high PA levels.

We were not able to directly explore mechanisms in these analyses. It is plausible that biomedical risk factors such as body mass index, blood pressure and cholesterol are on the intermediate pathway linking PA and CVD [16], thus we did not adjust for these factors in our models. The mechanisms accounting for the associations between PA and different CVD outcomes may differ, although this remains poorly understood. A key strength was the ability to sub-type major CVD deaths in sufficient numbers over an extended follow-up, together with our validated exposure measure. Limitations include the possibility of residual confounding as we lacked complete data on other lifestyle variables (sedentary behaviour, diet) and more detailed socioeconomic factors. Reverse causation is also a concern in analyses of PA and disease outcomes although the results remained unchanged when existing cases of CVD at baseline were removed. We were unable to obtain linkages for participants that emigrated through follow-up, thus they would have been excluded from the analytic sample. This reflects a very small proportion of the cohort and is unlikely to have influenced our results. However, 10.2% of the cohort did not consent to linkage. Participants not consenting to linkage were slightly older (60 vs. 58 years, *p* < 0.001), more likely to come from manual occupations (27.9 vs. 23.8%), and be physically inactive in leisure time (67.0 vs. 61.8%), although there were no differences in prevalence of CVD at baseline (8.9 vs. 9.0%) compared to the analytic sample. Thus, our analytic sample displayed a more favourable profile that may not have been truly representative of the general population. Given that only ~ 3.4% of the sample undertook regular strength training, our PA variable combined aerobic activity and strength exercises. Aerobic exercise, however, was more strongly associated with protection from CVD than strength training in previous work from this cohort study [17]. Given the lack of events for some outcomes it was challenging to undertake detailed investigation on the shape of the association.

In summary, physical activity was associated with reduced risk of seven major CVD death causes. All types of PA may contribute to CVD prevention, and protective benefits were apparent even at levels of activity below the current recommendations.

## Electronic supplementary material

Below is the link to the electronic supplementary material.

Supplementary material 1 (DOCX 20 kb)
