# Supplementary material for: Association between physical activity and sub-types of cardiovascular disease death causes in a general population cohort
Source: Eur J Epidemiol. 2018 Nov 11;34(5):483–7. doi: 10.1007/s10654-018-0460-2 (PMC6456476; doi:10.1007/s10654-018-0460-2)
Supplement: Supplementary file 1 — Supplementary material 1 (DOCX 20 kb) [file 10654_2018_460_MOESM1_ESM.docx]

**Online supplement**

Table e1. Associations between physical activity and different CVD death endpoints in participants aged 40 yrs and above in Health Survey for England/Scottish Health Survey (n=65,093).

| Outcome |  | **Meeting guidelines†** | | | | **Quintiles of *total* physical activity (MET-h-wk)** | | | | |
| --- | --- | --- | --- | --- | --- | --- | --- | --- | --- | --- |
|  |  | Inactive  n= 40,413 | Insufficient  n=13,121 | Sufficient  n=6826 | High  n=4751 | <1.64  n=14819 | 1.65 – 9.37  n=15096 | 9.38 – 19.30  n=12281 | 19.31 – 37.60  n=11939 | >37.6  n=10951 |
| All CVD deaths | Events | 2622 | 272 | 86 | 70 | 1416 | 679 | 394 | 323 | 238 |
|  | HR (95% CI) | 1.0 (ref) | 0.64 (0.56, 0.72) | 0.55 (0.45, 0.69) | 0.61 (0.48, 0.77) | 1.0 (ref) | 0.68 (0.62, 0.75) | 0.58 (0.52, 0.65) | 0.52 (0.45, 0.59) | 0.50 (0.43, 0.57) |
| Acute myocardial infarction | Events | 505 | 58 | 20 | 21 | 247 | 155 | 74 | 64 | 64 |
|  | HR (95% CI) | 1.0 (ref) | 0.62 (0.47, 0.83) | 0.62 (0.40, 0.97) | 0.80 (0.51, 1.26) | 1.0 (Ref) | 0.82 (0.67, 1.02) | 0.57 (0.44, 0.75) | 0.52 (0.39, 0.59) | 0.66 (0.50, 0.89 |
| Chronic ischaemic heart disease | Events | 764 | 73 | 28 | 16 | 429 | 186 | 115 | 78 | 73 |
|  | HR (95% CI) | 1.0 (ref) | 0.59 (0.46, 0.76) | 0.61 (0.42, 0.89) | 0.45 (0.28, 0.75) | 1.0 (Ref) | 0.64 (0.53, 0.76) | 0.58 (0.47, 0.71) | 0.42 (0.22, 0.53) | 0.49 (0.38, 0.64) |
| Pulmonary heart disease | Events | 71 | 10 | 4 | 2 | 43 | 12 | 11 | 12 | 9 |
|  | HR (95% CI) | 1.0 (ref) | 0.81 (0.41, 1.60) | 0.94 (0.34, 2.62) | 0.70 (0.17, 2.91) | 1.0 (Ref) | 0.33 (0.17, 0.63) | 0.43 (0.22, 0.85) | 0.51 (0.26, 1.00) | 0.48 (0.22, 1.07) |
| Arrhythmias | Events | 62 | 5 | 2 | 1 | 30 | 20 | 10 | 8 | 2 |
|  | HR (95% CI) | 1.0 (ref) | 0.48 (0.14, 1.20) | 0.52 (0.12, 2.15) | 0.33 (0.10, 2.38) | 1.0 (Ref) | 0.96 (0.53, 1.76) | 0.68 (0.32, 1.43) | 0.49 (0.20, 1.16) | 0.18 (0.04, 0.76) |
| Heart failure | Events | 218 | 29 | 7 | 5 | 135 | 61 | 21 | 28 | 14 |
|  | HR (95% CI) | 1.0 (ref) | 0.90 (0.61, 1.34) | 0.67 (0.31, 1.43) | 0.62 (0.25, 1.52) | 1.0 (Ref) | 0.68 (0.50, 0.93) | 0.35 (0.52, 0.96) | 0.51 (0.33, 0.78) | 0.35 (0.20, 0.63) |
| Cerebrovascular | Events | 545 | 54 | 17 | 17 | 285 | 133 | 102 | 71 | 42 |
|  | HR (95% CI) | 1.0 (ref) | 0.65 (0.49, 0.87) | 0.56 (0.34, 0.93) | 0.82 (0.50, 1.34) | 1.0 (Ref) | 0.69 (0.59, 0.86) | 0.82 (0.62, 1.04) | 0.64 (0.49, 0.84) | 0.53 (0.38, 0.75) |
| Aneurysm/ peripheral vascular | Events | 210 | 19 | 2 | 4 | 107 | 51 | 34 | 26 | 17 |
|  | HR (95% CI) | 1.0 (ref) | 0.63 (0.39, 1.01) | 0.19 (0.10, 0.76) | 0.49 (0.18, 1.31) | 1.0 (Ref) | 0.76 (0.54, 1.07) | 0.77 (0.51, 1.15) | 0.65 (0.42, 1.02) | 0.54 (0.34, 0.93) |

* Hazard ratio (HR) adjusted for age, sex, smoking, social occupational group, chronic illnesses, psychological distress.

†Reference category corresponds to participants not undertaking any MVPA; Insufficient activity are active but not meeting the current PA guidelines (150 min/wk moderate or 75 min/wk vigorous PA); Sufficient activity are those meeting the basic guidelines (150 min/wk moderate or 75 min/wk vigorous PA); High activity are those exceeding guidelines (more than 300 min/wk MVPA).

**Table e2.** Associations between physical activity MET-h-wk and different CVD death endpoints stratified into participants with (n=5,962) and without (n=59,131) CVD at baseline.

| Outcome |  | **Participants with CVD at baseline** | | | | | **Participants without CVD at baseline** | | | | |
| --- | --- | --- | --- | --- | --- | --- | --- | --- | --- | --- | --- |
|  |  | <1.64  n=2876 | 1.65 – 9.37  n=1256 | 9.38 – 19.30  n=708 | 19.31 – 37.60  n=619 | >37.6  n=505 | <1.64  n=11943 | 1.65 – 9.37  n=13840 | 9.38 – 19.30  n=11573 | 19.31 – 37.60  n=11320 | >37.6  n=10446 |
| All CVD deaths | Events | 524 | 187 | 83 | 56 | 48 | 892 | 492 | 311 | 267 | 190 |
|  | HR (95% CI) | 1.0 (ref) | 0.83 (0.70, 0.98) | 0.62 (0.49, 0.79) | 0.49 (0.37, 0.65) | 0.61 (0.45, 0.82) | 1.0 (ref) | 0.67 (0.60, 0.75) | 0.61 (0.53, 0.69) | 0.56 (0.48, 0.64) | 0.51 (0.43, 0.60) |
| Acute myocardial infarction | Events | 110 | 53 | 18 | 18 | 18 | 137 | 102 | 56 | 46 | 46 |
|  | HR (95% CI) | 1.0 (Ref) | 0.96 (0.68, 1.36) | 0.55 (0.33, 0.92) | 0.59 (0.35, 1.00) | 0.85 (0.50, 1.42) | 1.0 (Ref) | 0.85 (0.65, 1.11) | 0.65 (0.47, 0.89) | 0.56 (0.40, 0.79) | 0.71 (0.50, 1.00) |
| Chronic ischaemic heart disease | Events | 183 | 52 | 34 | 17 | 15 | 246 | 134 | 81 | 61 | 58 |
|  | HR (95% CI) | 1.0 (Ref) | 0.69 (0.50, 0.94) | 0.74 (0.51, 1.07) | 0.43 (0.26, 0.71) | 0.54 (0.31, 0.93) | 1.0 (Ref) | 0.67 (0.54, 0.83) | 0.57 (0.44, 0.74) | 0.46 (0.34, 0.61) | 0.54 (0.40, 0.73) |
| Heart failure | Events | 46 | 18 | 4 | 6 | 2 | 89 | 43 | 17 | 22 | 12 |
|  | HR (95% CI) | 1.0 (Ref) | 0.91 (0.52, 1.60) | 0.31 (0.11, 0.88) | 0.56 (0.23, 1.34) | 0.28 (0.10, 1.20) | 1.0 (Ref) | 0.64 (0.44, 0.93) | 0.37 (0.22, 0.64) | 0.52 (0.32, 0.85) | 0.39 (0.21, 0.74) |
| Cerebrovascular | Events | 94 | 29 | 12 | 8 | 4 | 191 | 104 | 90 | 63 | 38 |
|  | HR (95% CI) | 1.0 (Ref) | 0.79 (0.51, 1.21) | 0.60 (0.33, 1.12) | 0.48 (0.23, 1.00) | 0.37 (0.13, 1.04) | 1.0 (Ref) | 0.70 (0.54, 0.89) | 0.90 (0.69, 1.18) | 0.70 (0.52, 0.95) | 0.59 (0.40, 0.85) |
| Aneurysm/ peripheral vascular | Events | 36 | 15 | 8 | 4 | 5 | 71 | 36 | 26 | 22 | 12 |
|  | HR (95% CI) | 1.0 (Ref) | 1.13 (0.61, 2.10) | 1.15 (0.52, 2.52) | 0.73 (0.25, 2.09) | 1.25 (0.47, 3.29) | 1.0 (Ref) | 0.67 (0.44, 1.02) | 0.70 (0.44, 1.12) | 0.64 (0.39, 1.06) | 0.42 (0.22, 0.82) |

* Hazard ratio (HR) adjusted for age, sex, smoking, social occupational group, chronic illnesses, psychological distress.
